# Testing the Efficacy of a Scalable Telephone-Delivered Guided Imagery Tobacco Cessation Treatment: Protocol for a Randomized Clinical Trial

**DOI:** 10.2196/48898

**Published:** 2023-06-23

**Authors:** Judith S Gordon, Julie S Armin, Peter Giacobbi Jr, Chiu-Hsieh Hsu, Kari Marano, Christine E Sheffer

**Affiliations:** 1 College of Nursing University of Arizona Tucson, AZ United States; 2 Department of Family and Community Medicine University of Arizona Tucson, AZ United States; 3 College of Applied Human Sciences West Virginia University Morgantown, WV United States; 4 School of Public Health West Virginia University Morgantown, WV United States; 5 Mel and Enid Zuckerman College of Public Health University of Arizona Tucson, AZ United States; 6 Department of Health Behavior Roswell Park Comprehensive Cancer Center Buffalo, NY United States

**Keywords:** tobacco, smoking, treatment, integrative health, guided imagery, behavior change, telephone, mobile phone

## Abstract

**Background:**

Tobacco use continues to be a leading preventable cause of death and disease in the United States, accounting for >480,000 deaths each year. Although treatments for tobacco use are effective for many, there is substantial variability in outcomes, and these approaches are not effective for all individuals seeking to quit smoking cigarettes. New, effective therapeutic approaches are needed to meet the preferences of people who want to stop smoking. Guided imagery (GI) is a mind-body technique that involves the guided visualization of specific mental images, which is enhanced with other sensory modalities and emotions. Preliminary evidence provides initial support for the use of GI as a treatment for cigarette smoking. Meta-analyses have shown that standard treatment for cigarette smoking delivered over the telephone via quitlines is effective. A telephone-based intervention that uses GI might provide another effective treatment option and increase the reach and effectiveness of quitlines.

**Objective:**

This study aims to test the efficacy of *Be Smoke Free*, a telephone-delivered GI treatment for smoking cessation.

**Methods:**

This multisite randomized clinical trial (RCT) will compare a novel telephone-delivered GI tobacco cessation treatment with a standard evidence-based behavioral treatment. The study will be conducted over 5 years. In phase 1, we refined protocols and procedures for the New York State and West Virginia sites for use in the RCT. During phase 2, we will conduct an RCT with 1200 participants: 600 (50%) recruited via quitlines and 600 (50%) recruited via population-based methods. Participants will be randomly assigned to either the GI condition or the behavioral condition; both treatments will be delivered by trained study coaches located at the University of Arizona. Assessments will be conducted at baseline and 3 and 6 months after enrollment by University of Arizona research staff. The primary outcome will be self-reported 30-day point prevalence abstinence 6 months after enrollment. Secondary outcomes include biochemically verified 7-day point prevalence abstinence 6 months after enrollment.

**Results:**

Recruitment in West Virginia and New York began in October 2022. As of March 31, 2023, a total of 242 participants had been enrolled. Follow-up assessments began in November 2022. As of March 31, 2023, of the 118 eligible participants, 97 (82.2%) had completed the 3-month assessment, and 93% (26/28) of eligible participants had completed the 6-month assessment. Biochemical verification and qualitative interviews began in April 2023. Recruitment will continue through 2025 and follow-up assessments through 2026. Primary results are expected to be published in 2027.

**Conclusions:**

The *Be Smoke Free* study is a first-of-its-kind RCT that incorporates GI into telephone-based tobacco cessation treatment. If successful, *Be Smoke Free* will have substantial benefits for the long-term health of people who use tobacco across the United States.

**Trial Registration:**

ClinicalTrials.gov NCT05277831; https://clinicaltrials.gov/ct2/show/NCT05277831

**International Registered Report Identifier (IRRID):**

PRR1-10.2196/48898

## Introduction

### Background

Tobacco use continues to be a leading preventable cause of death and disease in the United States, accounting for >480,000 deaths each year, including 41,000 deaths from secondhand smoke exposure [1,2]. The annual economic cost of tobacco use in the United States was US $332 billion in 2014 and increases every year [2,3]. Moreover, tobacco use aligns with other systemic health inequities in the United States [1,4]. The prevalence of tobacco use among Medicaid beneficiaries and individuals of lower socioeconomic status is nearly twice that among the general population [1,4]. Importantly, however, tobacco cessation results in a nearly universal improvement in health.

Although evidence-based treatments for tobacco use are effective for many, there are multiple limitations, including a substantial variability in outcomes and limited reach. Current evidence-based approaches are not effective for every individual seeking to stop smoking [2,5]. Quitlines have made standard cognitive behavioral treatment widely accessible, but they reach fewer than 1% of the individuals who smoke cigarettes annually. In addition, evidence suggests that at least some of the individuals who smoke do not wish to engage in *standard* cognitive behavioral treatment and would prefer a more holistic or integrative approach (ie, combining complementary or alternative and standard treatments) [6-9]. New therapeutic approaches are needed to attract, engage, and retain individual tobacco users in treatment for smoking cessation and ultimately increase the number of individuals who achieve long-term abstinence from tobacco use.

A large proportion of the American population uses integrative health approaches, including mind-body techniques, in addition to, or instead of, conventional treatment [5]. Guided imagery (GI) is a mind-body technique that involves the visualization of mental images. GI is an alternative way to develop effective strategies to quit smoking, such as developing awareness and directing attention to one’s thoughts, feelings, situational cues or triggers, and urges. GI can effectively assist individuals to quit or reduce smoking [6-8]. A randomized controlled trial (RCT) with 779 adult smokers compared the use of bupropion versus brief psychotherapy that included GI intended to enhance self-management, decidedness, assertiveness, self-determination, and self-assurance [8]. Intent-to-treat analysis showed 12-month abstinence rates of 39.1% and 12.3% for the psychotherapy and bupropion groups, respectively [8]. In another RCT, 33 adults were taught mindfulness and GI and encouraged to practice at home [9]. The results showed that home practice predicted reduced cigarette use over 4 weeks [9]. In a pilot RCT, a GI program for smoking cessation was found to be feasible and improved intermediate abstinence measures [10]. In a small trial that compared education and counseling with education and counseling plus GI training, abstinence rates were 12% and 26%, respectively [7]. Finally, our teams’ preliminary evidence provides strong support for the use of GI for smoking cessation [11,12]. These findings support the potential efficacy of GI for smoking cessation. In addition, we are proposing to deliver a GI intervention in a low-cost highly scalable way (ie, by telephone). To date, no studies have established the efficacy of an integrative GI tobacco cessation treatment delivered by telephone.

Several meta-analytic reviews have shown that proactive telephone-based tobacco cessation services are an effective way to deliver treatment for tobacco dependence [13-15]. Telephone quitlines are efficient, centralized, and a highly scalable way for individuals to access tobacco treatment in all 50 states plus the District of Columbia, Guam, and Puerto Rico. Quitlines are the largest tobacco treatment network in the United States [16,17]. Researchers have demonstrated the cost-effectiveness of telephone quitlines in the United States and Europe over the past 25 years [3,13,15,18]. However, quitlines have limited reach into the population of people who use tobacco in the United States. According to a recent study, most US quitlines reach only 1.1% of this population [19]. This situation has prompted many state sponsors to try to improve their reach by increasing the types of evidence-based treatment offered to tobacco users. Evidence-based integrative treatment approaches that are appealing to those individuals not interested in standard behavioral approaches may increase the reach and effectiveness of quitlines. Using telephone technology enables access to a wide range of people who smoke because it does not rely on having an internet connection or competence in using more advanced technology. According to a 2019 survey [20], about 96% of Americans own a mobile phone of some kind; thus, our intervention is highly accessible and scalable.

### Objectives

The objective of this RCT is to test the efficacy of *Be Smoke Free*, a telephone-delivered integrative GI treatment for smoking cessation compared with a standard, evidence-based behavioral treatment. The specific aims and hypotheses of the study are described in the following subsections.

#### Aim 1

We will test the efficacy of a telephone-delivered integrative GI tobacco cessation treatment versus an evidence-based behavioral treatment on self-reported 30-day abstinence 6 months after enrollment (T2). Secondary outcomes are self-reported and biochemically verified 7-day point prevalence smoking abstinence at T2.

#### Hypothesis 1

Participants in the GI condition (GIC) will demonstrate 10% higher quit rates than those in the behavioral condition (BC).

#### Aim 2

We will conduct dose-response analyses on the effect of GIC adherence (measured by self-reported minutes of intervention use per week, the number of times GI skills practiced per week, the number of sessions attended, and coach-rated participant engagement in sessions) on abstinence at 6 months. We will also examine effects of the GIC and BC on tobacco use for those participants who do not report abstinence at 6 months.

#### Hypothesis 2A

GIC participants who are more adherent to the intervention will have higher rates of abstinence.

#### Hypothesis 2B

GIC and BC participants who do not report abstinence will report statistically significant reductions in tobacco use.

#### Aim 3

We will conduct subgroup analyses of moderators (eg, recruitment method, location, sex, race and ethnicity, and the level of tobacco dependence) on tobacco cessation outcomes at 6 months and explore subgroup differences in participants using a mixed methods approach (eg, surveys and in-depth interviews).

### Project Impact

The project has the potential for high overall impact. Should this approach become readily available to quitlines as an evidence-based treatment, it will provide a sustainable and powerful alternative to the traditional treatment options available throughout the United States. There have been few innovations in quitline treatment. Thus, increasing the treatment options might also serve to increase the reach of quitlines into the tobacco-using population.

## Methods

### Overview

This 5-year, 2-group RCT will test the efficacy of the integrative GI treatment versus an evidence-based behavioral treatment. Participants will be recruited from 3 states (Arizona, New York State, and West Virginia). We will randomize 1200 participants to either the GIC or the BC: 600 (50%) participants will be recruited through the quitlines (n=200, 33.3%, per quitline) and 600 (50%) through population-based methods (eg, social media, earned media, local organizations, and health care providers) to ensure that we obtain a representative sample of people who smoke. Assessments will occur at baseline (T0), 3 months after enrollment (T1), and T2. The research team will deliver the GIC and BC protocols, coordinate and conduct all assessments, and perform all data analyses at the University of Arizona. The primary outcome will be self-reported 30-day abstinence at T2. Secondary outcomes will be self-reported and biochemically verified (expired carbon monoxide [CO]) 7-day point prevalence abstinence at 6 months.

This study is being conducted in 2 phases: during phase 1, we refined protocols and procedures for the New York State and West Virginia sites for use in the RCT. During phase 2, we are conducting the RCT.

### Phase 1: Revisions to Protocols and Procedures

We worked with our collaborating quitlines to revise procedures for recruitment, screening, data transfer, and reporting for half of our sample. Each of our participating sites provides different services and uses different procedures for screening callers. This required us to tailor our study recruitment, screening protocols, and procedures for each site. We also worked with each quitline to identify existing networks (eg, health departments and community organizations) to add to our population-based recruitment methods for each state. In addition, we tailored the methods for transferring participant data from each quitline to our project staff in a Health Insurance Portability and Accountability Act (HIPAA)–compliant and secure manner. Finally, we worked with each site to determine the process for providing regular reports on client contacts and tracking callers transferred to study staff. Noneligible callers who are transferred by project staff to our study will be transferred back to their respective quitlines so that they may return for quitline services before study completion. We worked with staff at each site to learn about their unique structures and tailor all study processes and materials to fit both the needs of the quitline and our study. Half of our sample will be recruited through the quitlines.

We will use population-based methods (eg, social media paid advertising, earned media, and community contacts) for the other half of the sample. The materials and methods created during the feasibility trial were designed for use in Arizona [21]. Therefore, they were modified for use in the other states participating in this study. These states serve different demographics of tobacco users who may also need to be reached in different ways (eg, rural vs urban and print vs web based). We worked with our collaborators in each state to identify changes that needed to be made to the text and graphics in our recruitment advertisements and materials (eg, wording and images for advertisements placed on social media and different photographs representing tobacco users in each state for use in print materials). We convened a community advisory board (CAB) that included key informants from each state (eg, members of community health organizations identified by our collaborators) to obtain information using semistructured questionnaires. Interviews or group interviews were recorded and transcribed. The study team analyzed the comments and developed a plan for refining the recruitment methods and materials for each state.

In consultation with our collaborating quitlines, we made minor refinements to our study protocols and corresponding participant materials to address the specific needs of people who use tobacco in different states; for example, although all participants will identify their primary tobacco use as cigarette smoking, a proportion will also use other tobacco products (eg, e-cigarettes or vaping products and smokeless tobacco). The use of these other tobacco products varies widely by state. The prevalence of smokeless tobacco use is very low in Arizona but much higher in West Virginia (2.8% vs 8.9%, respectively) [22]. Therefore, we have included additional information in the protocols to address secondary smokeless tobacco use. On the basis of the information and feedback we received from each participating site, we made final changes to our protocols, which were programmed into REDCap (Research Electronic Data Capture; Vanderbilt University). Using REDCap allows our coaches to consistently deliver the protocol for each condition across participants and collect data on each session. For this RCT, we used the same REDCap system developed under the feasibility trial.

On the basis of changes to the study protocols, we made corresponding refinements to the coach training program and coach materials. Information was added regarding regional differences in smoking and other tobacco use patterns, culturally responsive counseling for specific racial and ethnic groups, issues specific to rural versus urban tobacco users, and other state-specific differences that the coaches may encounter when working with participants across our participating states.

We hired 4 coaches (2 for each condition) to deliver one of the study protocols at the University of Arizona. The training of the intervention coaches included a 2-part workshop, individual and group exercises designed to provide practical experience that resulted in competency to deliver the protocols and achieve a positive therapeutic alliance, and ongoing supervision designed to solve problems and maintain implementation fidelity. The workshops for both conditions involved the cognitive and motivational underpinnings of each program. The BC workshop focused solely on behavioral strategies for motivating and assisting tobacco users to quit and the use of nicotine replacement therapy for minimizing withdrawal symptoms. The GIC workshop included all content in the BC workshop plus the use of GI, including how to create vivid and evocative GI scripts and record them as audio files for participants assigned to this condition.

### Phase 2: RCT

#### Study Design

**Figure 1:**
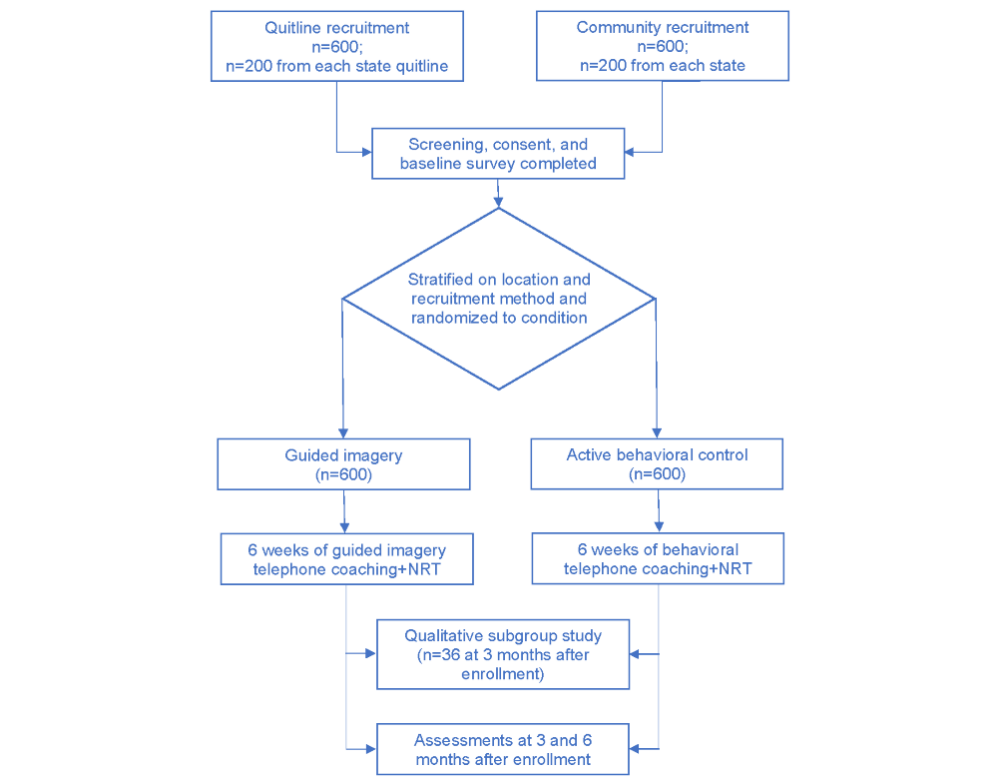
Study design. NRT: nicotine replacement therapy.

This 2-group RCT will test the efficacy of the integrative GIC versus the evidence-based BC. Participants will be recruited from 3 states (Arizona, New York State, and West Virginia). We will randomize 1200 participants to either the GIC or the BC. Of these 1200 participants, 600 (50%) will be recruited through the quitlines (n=200, 33.3%, per quitline) and 600 (50%) through population-based methods (eg, social media, earned media, local organizations, and health care providers) to ensure that we obtain a representative sample of smokers (Figure 1). Assessments will occur at T0, T1, and T2. The research team will deliver the intervention and control protocols, coordinate and conduct all assessments, and perform all data analyses at the University of Arizona.

#### RCT Protocol

The study protocol was adapted from our existing protocol [12,23]. Participant recruitment occurs during years 2 to 4 of the project, after coaches are trained and competent in the GIC and BC protocols. All screening, enrollment, and treatment activities are conducted by telephone, email, and SMS text message by research staff at the University of Arizona.

Recruitment occurs through quitlines and population-based activities. Quitline staff prescreen potential participants and describe the study to callers. Quitline staff collect eligible and interested callers’ contact information, including their telephone number, and transfer these data to project staff located at the University of Arizona. For population-based activities, potential participants recruited through population-based methods contact project staff.

For both recruitment methods, project staff contact interested callers within 1 business day. Project staff provide a detailed description of the study. Callers who are interested in participating are screened again for eligibility by project staff. Staff then consent eligible callers and administer the T0 survey or send survey links (REDCap) by SMS text message or email to participants who choose to complete the forms themselves. Those participants who complete the T0 survey are randomly assigned to the GIC or the BC via REDCap. Noneligible or noninterested callers are transferred back to their state quitline. Randomized callers are contacted by their study coach within 2 business days. Once callers complete their first session, they are considered enrolled participants in the study. All coaches are located at the University of Arizona. Participants in each condition receive 6 sessions, lasting from 10 to 60 minutes, depending on the condition and session topic.

All session activities and interactions with participants are managed and recorded in REDCap. Coach competencies and protocol fidelity are routinely monitored by the investigators. Process data (ie, coach notes and in-depth interviews) will be collected during the 6-week program and at T1.

Participants are assessed via REDCap or telephone (for nonresponders) at T1 and T2 by project staff. A subsample of participants (36/1200, 3%) will be recruited to participate in surveys and in-depth interviews to explore subgroup differences in intervention use, response, and satisfaction. Participants will receive up to US $100 (in gift cards) for their efforts: US $10 for completing the T0 assessment, US $15 for the 3-month assessment, US $25 for the 6-month assessment, and US $50 for the biochemical verification. Participants chosen for the qualitative interviews will receive a US $25 gift card.

#### Participant Recruitment, Eligibility, Enrollment, and Randomization

##### Recruitment Methods

Participants are recruited from two populations: (1) individuals who contact one of our collaborating quitlines; or (2) individuals who smoke in Arizona, New York State, or West Virginia. Different recruitment methods will be used to recruit participants from each population. For the quitline population, we monitor quitline recruitment—an increase or decrease in the number of days per week that we recruit based on the volume of incoming calls. For population-based recruitment, we send out press releases describing the study, post on social media sites, network with our contacts in each state, and place paid advertisements, as needed. We monitor recruitment weekly and make adjustments to recruitment procedures to ensure that we are meeting our recruitment goals. We do not foresee any problems recruiting our sample according to the timeline. However, we have an additional 3 months in which we can extend our recruitment period if needed.

##### Eligibility Criteria

The inclusion criteria are as follows: primary tobacco use is smoking; smoke daily for the last 30 days; aged at least 18 years; speak English; have a smartphone with SMS text messaging; do not take antipsychotic medications or experience symptoms of psychosis; have not received quitline coaching for the previous 12 months; agree to telephone coaching and SMS text message reminders; can download an mp3 or mp4 file; live in Arizona, New York State, or West Virginia; no current use of any tobacco cessation program or medication; and only 1 participant per household.

##### Enrollment

Potential participants are screened by trained study staff over the telephone or via REDCap. Eligible participants complete the T0 survey and informed consent over the telephone or on REDCap. Participants download the consent form from REDCap or request that study staff send them a hard copy. Study staff send condition-specific program materials to randomized participants. Ineligible callers are transferred back to the quitline in their state of residence using a *warm handoff* (ie, study staff call the quitline, talk to enrollment staff, and then transfer the caller directly to the quitline enrollment staff) or a HIPAA-compliant electronic process (eg, encrypted email).

##### Randomization

Participants are stratified by state and method of recruitment (ie, quitline or community) and then randomized automatically in REDCap using a randomization table created by the study biostatistician. Statistical software was used to create a randomized-block allocation to the GIC or the BC, stratified by recruitment method and location.

#### Measures

##### Overview

All measures and the time points at which they are administered are displayed in Table 1. All these measures have been used in our previous work [11,12,23-25].

**Table 1:** Measures by time point for both conditions.

	T0^a^	T1^b^	T2^c^
Demographics	✓		
Tobacco use	✓	✓	✓
Dependence	✓	✓	✓
Cravings	✓	✓	✓
Self-efficacy	✓	✓	✓
Expectancy and credibility	✓	✓	✓
VVIQ^d^	✓	✓	✓
Biochemical verification (expired CO^e^)			✓
Consumer satisfaction		✓	
Sociotechnical outcomes		✓	

^a^T0: baseline.

^b^T1: 3 months after enrollment.

^c^T2: 6 months after enrollment.

^d^VVIQ: Vividness of Visual Imagery Questionnaire.

^e^CO: carbon monoxide.

##### Demographics

At T0, we will collect sex, age, race and ethnicity, level of education, insurance, marital status, smokeless tobacco use, cannabis use, chronic health conditions, and prior use of guided mental imagery.

##### Tobacco Use

At T0, T1, and T2, we will collect tobacco use status using a series of questions that have been standardized and used in previous studies [25-27] and the level of dependence using the Fagerström Tolerance Nicotine Dependence Scale [28].

##### Self-Efficacy for Quitting

We will measure self-efficacy for quitting smoking with an abbreviated version of the Condiotte and Lichtenstein Confidence Questionnaire [29].

##### Cravings

All participants will be asked to rate their experience with withdrawal symptoms and cravings using the Nicotine Dependence Syndrome Scale [30] at each follow-up assessment.

##### Expectancies and Credibility

We will include items adapted from the Borkovec and Nau Treatment Credibility Scale [31] and used in our completed GI mobile app study to measure expectancies and perceived credibility of GI for smoking cessation [11,23]. We will also adapt this measure to assess for expectancies and perceived credibility of the behavioral program used in the BC.

##### Consumer Satisfaction Measure

At T1, participants will complete a consumer satisfaction survey that we have used in our previous research [25,32] and consists of up to 11 items (using a 5-point Likert scale), measuring overall satisfaction with the program in each study condition, perceived usefulness and relevance of the information, likeability, the level of interest, and the ease of use, as well as whether they would recommend the program to others.

##### Intervention Adherence

Adherence will be measured by a composite of self-reported minutes of intervention use per week, the number of times skills practiced per week, the number of sessions attended, and coach-rated participant engagement in sessions.

##### Imagery Vividness and Sociotechnical and Ethical Outcomes

At T0, T1, and T2, we will assess participants’ perceptions about the effectiveness of GI for tobacco cessation and their vividness of visual imagery using the Vividness of Visual Imagery Questionnaire, which we have adapted to include images of smoking [33]. At T1, we will examine participants’ use of GI during the RCT and their perception of the GI program’s *fit* in their lives through semistructured in-depth interviews [34,35].

##### Biochemical Verification of Tobacco Abstinence

All participants who self-report 7-day abstinence at the 6-month assessment will be selected for biochemical verification. We will (1) use enhanced collection procedures, including contact with participants via telephone before the assessment and via video call during the assessment if participants request assistance; (2) provide an iCO Smokerlyzer expired CO monitor (Bedfont Scientific Limited) to each participant who reports abstinence, a procedure that has been used successfully in previous remote collection studies and has high accuracy [36-38]; and (3) provide an incentive of US $50 for the completion of the biochemical assessment.

##### Biomarkers

Expired CO will be measured among smokers using iCO Smokerlyzer personal CO monitors. A monitor will be sent to each participant who reports abstinence at the 6-month follow-up assessment. Each participant will exhale into the monitor according to instructions. Readings are measured in parts per million. A cutoff of <6 parts per million is recommended [39]. The results will be transmitted electronically to the research team.

##### Collection Procedures

Project staff will contact participants who report abstinence at the 6-month assessment via SMS text message, telephone, or email to schedule a HIPAA-compliant encrypted video call during which biochemical verification will be completed. On the scheduled day of assessment, project staff will verify the participant’s identity through the confirmation of their name and face from a photograph ID. During this session, project staff will provide step-by-step instructions for completing biochemical verification.

#### Outcomes

Our primary outcome is self-reported 30-day abstinence as measured at the 6-month assessment. Secondary outcomes include self-reported 7-day abstinence as well as biochemically verified 7-day abstinence at the 6-month assessment and, for nonquitters, cigarettes smoked per day.

We will also examine the effect of adherence to the GI on cessation outcomes, conduct subgroup analyses of moderators (eg, recruitment method, location, sex, race and ethnicity, and level of dependence) on tobacco cessation outcomes at 6 months, and use a mixed methods approach (eg, surveys and in-depth interviews) to explore subgroup differences in GI adherence and cessation outcomes.

#### Qualitative Subgroup Study

Subsample participants (36/1200, 3%) will be recruited after a preliminary analysis of the T1 data and based on the following criteria: recruitment method, location, sex, race and ethnicity, and abstinence status. After a quantitative analysis of the subgroups, the team will identify 36 participants through purposive sampling (eg, identifying participants who were recruited through community methods). Only those participants who indicate an interest in participating in in-depth interviews at T1 will be invited to participate in in-depth interviews via telephone, email, or SMS text message. One-hour telephone interviews will be conducted by a member of the study team, who will use a semistructured interview guide to explore the following aspects: program use during the RCT (eg, how did the technology they used influence completion of coaching calls?), their perceptions of program effectiveness for tobacco cessation (eg, how does GI work to help them stop smoking?), and their perception of the program’s *fit* in their lives (eg, how does GI integrate or not integrate with their lifestyle?).

#### Power and Sample Size

A sample size of 1200 participants would yield 90% power to detect a difference in smoking cessation rates between the arms of 10%, assuming a control arm cessation rate of 0.30, a 20% dropout, and a noncontinuity-corrected chi-square test at a 2-tailed significance level of 5%. We believe that 10% is a clinically significant difference with positive public health implications. Although the control arm had a higher cessation rate in our pilot study (50%) [19], we believe that this rate was unusually high. Furthermore, the rates were self-report, not biochemically verified, which are estimated to be 10% to 50% lower [39]. It was also high compared with quitline cessation rates, which are closer to 30% to 40% [15,40].

This sample size also yields 90% power to detect small-to-medium standardized effect sizes for continuous secondary outcomes of Cohen *d*=0.28, using a Bonferroni correction to account for multiplicity (20 tests), assuming a 2-sided 2-sample *t* test. Aim 3 will investigate effect modification, which requires approximately 4 times the sample size (or, equivalently, results in a larger detectable effect size) [41]. Assuming that a subgroup of interest makes up 50% of the sample, we have 90% power to detect a difference in cessation rates of 0.141 and 80% to 90% power to detect standardized effect sizes of 0.26 to 0.30, assuming no multiple testing correction. We recognize that the inputs to these calculations are estimates only.

Table 2 displays a sensitivity analysis, showing power for control arm cessation rates of 0.20 and 0.40, dropout rates of 20% to 40% (typical range for cessation trials), and differences of 0.10 to 0.15, showing ample power for different scenarios. These power calculations are conservative; simulation studies demonstrate that mixed effects models (the proposed analyses [Data Analysis subsection]) yield more power than chi-square and *t* tests [42,43].

**Table 2:** Sensitivity analysis (N=1200).

Dropouts, n^a^	Cessation rate: 0.20			Cessation rate: 0.40	
	Detectable difference: 0.10	Detectable difference: 0.15	Detectable difference: 0.10		Detectable difference: 0.15
240	0.95	0.99	0.88		0.99
360	0.92	0.99	0.83		0.99
480	0.87	0.99	0.77		0.98

^a^Dropout rate: 20% (n=240); 30% (n=360); 40% (n=480).

#### Data Analysis

##### Overview

This is a 2-arm parallel RCT where the primary outcome is self-reported 30-day abstinence at 6 months. Questionnaires will be scored according to developer instructions. In the case of missing items, where developers do not have explicit instructions, we will use a half-mean imputation rule, as outlined in Bell et al [44]. All analyses will be adjusted for the randomization stratification. The primary analysis will use a significance level of .05; other tests will use a Bonferroni correction for multiple comparisons to control the overall type I error at 5%. Demographics, including sex, will be described with means, SDs, ranges, and frequencies or proportions and explored as correlates for successful tobacco outcomes using appropriate regression models.

##### Aim 1

We will test the efficacy of a telephone-delivered GI tobacco cessation treatment versus an evidence-based behavioral treatment on self-reported 30-day abstinence from smoking at T2. Secondary outcomes include self-reported and biochemically verified 7-day point prevalence abstinence at T2.

##### Hypothesis 1

Participants in the GIC will have 10% higher quit rates than those in the BC.

Generalized linear mixed models with a binomial distribution and a logit link will be used to model smoking outcomes. Time will be used categorically to avoid model misspecification, and the indicators for the stratified variables (ie, location and recruitment method) will be included in the models as covariates to control for the study design variables. Comparisons between the intervention and control groups at 3 and 6 months will be carried out using contrasts within these models. Mixed models are robust to missing outcome data (with respect to bias) and consistent with an intention-to-treat analysis; in addition, they yield more power than chi-square and *t* tests [42,43,45-47]. Secondary outcomes measured over time, including self-reported abstinence, nicotine dependence, self-efficacy, and cravings, will also be analyzed with mixed models (linear for continuous outcomes and generalized linear with a logistic link for binary). Biochemically verified abstinence at 6 months will be analyzed with a logit regression model. Unadjusted and adjusted models will be fitted, with adjusted models including key covariates such as expectancy. Sensitivity analyses with respect to missing data will be undertaken if missing data rates for the primary outcome are >10% at the 6-month follow-up. Multiple imputation by chained equations using data that are associated with missingness, the outcome, or both will be included in the imputation model [16]. We will compare T0 characteristics of participants who drop out with those of participants with complete data.

##### Aim 2

We will conduct dose-response analyses on the effect of GIC adherence (measured by self-reported minutes of intervention use per week, the number of times GI skills practiced per week, the number of sessions attended, and coach-rated participant engagement in sessions) on abstinence at 6 months. We will also examine effects of the GIC and the BC on tobacco use for those participants who do not report abstinence at 6 months.

##### Hypothesis 2A

GIC participants who are more adherent will have higher rates of abstinence at 6 months.

##### Hypothesis 2B

Participants not reporting abstinence will report statistically significant reductions in tobacco use.

We will investigate a dose-response effect of intervention engagement using logistic regression for cessation at 6 months and linear regression for dependence, self-efficacy, and so on, with adherence. Mediation models using a causal framework will also be used [17]. We will compare self-reported tobacco use levels in GI participants who have not quit at 6 months with the control group using regression models and also investigate their changes from T0 use. Consumer satisfaction will be described with summary statistics.

##### Aim 3

We will conduct subgroup analyses of moderators (eg, recruitment method, location, sex, race and ethnicity, and the level of dependence) on tobacco cessation outcomes at 6 months and assess participants using a mixed methods approach (eg, surveys and in-depth interviews) for exploring subgroup differences.

Logistic regression models will be used to investigate effect modification of the intervention effect on abstinence at 6 months by various moderators. These regression models will include interaction terms, and if statistically significant, a stratified (by moderator) analysis will be performed and reported.

Qualitative interviews will be recorded, transcribed, and consensus coded for themes by 2 coders to guarantee intercoder reliability [48,49]. The team will document variation in the subgroups’ reported program use during the RCT, including perceptions of the program’s effectiveness and ease of use.

#### Project Timeline

This project will occur in 2 phases over 5 years. In phase 1 (year 1), we worked with our collaborators in New York State and West Virginia to tailor our protocols and procedures to their quitlines, refine our recruitment methods to each state’s smokers, refine all study (participant) materials to ensure that the demographics of each state are represented, and update our training materials to include state-specific data and issues that may be relevant to specific participant populations. We hired and trained all coaches to competency and user-tested all our systems and procedures. In phase 2, we will conduct the RCT. Recruitment, enrollment, T0 assessments, and the delivery of the intervention will occur during years 2 to 4. Follow-up assessments will occur during years 2 to 5, with data analyses and dissemination of the findings in year 5.

### Ethics Approval, Informed Consent, Data Protection, and Participation

This study has been reviewed and approved by the University of Arizona institutional review board (2103633455; principal investigator [PI]: JG). All participants complete an informed consent document either through REDCap or on the telephone with trained study staff. Potential participants may ask questions of study staff before signing the consent document. The consent document describes the study, expectations, risks, and benefits of study participation. All study data are housed in REDCap and University of Arizona Box Health. Both systems are encrypted and HIPAA compliant. Only trained study staff have access to participant data, and data are restricted to study staff by role on the project. Participants may receive up to US $125 for participating in all study activities. Participants receive US $10 for completing the T0 survey, US $15 for completing the 3-month survey, and US $25 for completing the 6-month survey. All participants who report 7-day abstinence at the 6-month assessment are asked to complete an expired CO test to biochemically verify abstinence. Participants who complete biochemical verification receive US $50. Some participants will be selected based on their responses to the 3-month survey and invited to participate in a semistructured interview. Participants who complete this interview will receive US $25.

## Results

The *Be Smoke Free* study was funded in September 2021 (National Center for Complementary and Integrative Health, R01AT011500; PI: JG). Recruitment started in August 2022, with a staggered start. The West Virginia and New York State quitlines and community recruitment were all active as of October 2022. As of March 31, 2023, a total of 242 participants had been enrolled in the study, surpassing our accrual goal of 240. The Arizona Smokers’ Helpline (ASHLine) contract was transferred to another vendor, and we lost this as a recruitment site. The New York State and West Virginia quitlines each agreed to recruit an additional 100 participants to compensate for the loss of 200 Arizona quitline participants.

Follow-up assessments began in November 2022. As of March 31, 2023, of the 118 participants who were eligible for the 3-month assessment, 97 (82.2%) completed the assessment, and 93% (26/28) of participants eligible for the 6-month assessment had completed it. Because of technical difficulties with the hardware and software, biochemical verification began in April 2023. Qualitative interviews began in April 2023. Recruitment will continue through 2025 and follow-up assessments through 2026. Primary results are expected to be published in 2027.

## Discussion

### Overview

Phase 1 of the study was conducted between October 1, 2021, and July 30, 2022, and completed successfully. The study team met weekly and updated the design, content, and formatting of the intervention quit booklets that were created for the pilot study. We also created recruitment materials, including digital and print media, featuring images representing various communities. We recruited 8 stakeholders from 3 states to form a CAB. We held general and state-specific CAB meetings to obtain feedback on recruitment and intervention materials, as well as recruitment plans and methods. We also updated and refined coach training materials, revised data collection materials from the pilot study, and created new data collection materials. We developed protocols for screening, contact attempts, biochemical verification, and other data collection procedures and programmed and tested them all in the REDCap database. We developed procedures for recording and storing coaching calls using new Zoom (Zoom Video Communications, Inc) technology and hired and trained 4 quit coaches and all research staff. We received institutional review board approval for all human participants study procedures and registered the study with ClinicalTrials.gov.

We began phase 2, conducting the RCT, in July 2022. We have experienced both challenges and successes in the first 6 months of the RCT. To date, we have recruited participants through the West Virginia and New York State quitlines and via study social media posts and posts from community partners in Arizona, New York State, and West Virginia. We designed and sent recruitment postcards to former New York State quitline clients, used display ads on Meta sites, advertised on radio stations in New York State and Arizona, and distributed rack cards and flyers to our CAB members and other contacts in the community (eg, physicians’ offices). We recruited our first participant in Arizona in July 2022, then opened recruitment in West Virginia in August 2022, and in New York State in September 2022. In September 2022, the ASHLine contract was transferred from the University of Arizona to National Jewish Health, and the Arizona Department of Health Services declined to collaborate on the rest of the study. The New York State Smokers’ Quitline agreed to increase recruitment to compensate for the loss of the ASHLine.

Potential participants who were referred by a quitline or found out about the study from community-based methods were contacted via telephone calls, emails, and SMS text messages and screened by study staff. Potential participants who met the eligibility criteria completed consent and T0 assessments on the web in REDCap and were randomized to 1 of 2 study conditions.

Randomized participants who scheduled and completed the first study visit (session 1) were considered *enrolled participants* in the study.

To ensure that we are meeting our goals and maintaining fidelity of all procedures, we review enrollment and recruitment progress at weekly study team meetings, monitor intervention fidelity in REDCap, and review call recordings on a monthly basis. Any discrepancies in fidelity are immediately corrected, and study personnel receive weekly feedback. Coaches attend individual monthly supervision sessions with the PI.

We are currently retaining >80% of the sample at 3 months and >75% at 6 months. This is similar to our pilot study [12] and compares favorably with other studies that are conducted entirely remotely [50]. If participants miss some data collection visits or drop out from the study after session 1, partial data will be captured for them. As indicated previously, mixed effects models will be used to model smoking outcomes. Mixed effects models have an inherent way to handle ignorable missing data mechanisms (ie, missing completely at random and missing at random), which can be accounted for based on the observed data. In addition, extensive efforts will also be made to maximize retention as well as minimize missing data, and reasons for dropping out and missing data will be recorded. If the missing data mechanism is suspected to be nonignorable (ie, missing not at random), a multiple imputation–based sensitivity analysis approach [51] will be used to evaluate the impact of missing data mechanisms on the intervention effect, where the sensitivity analysis parameter controls the missing mechanism, and the multiply imputed data will be analyzed using standard methods based on the established rules in Rubin [52] that account for uncertainty owing to missingness.

### Limitations

We experienced several challenges during the first year of the RCT. First, we lost one of our quitline partners. Despite many attempts to replace the ASHLine, we were unable to do so. Both the New York State and West Virginia quitlines agreed to compensate for our loss of the ASHLine. However, West Virginia experienced a drop-off in call volume and staffing shortages. Therefore, the New York State Smokers’ Quitline increased their recruitment to maintain accrual goals. Although the demographics of New York State are different from those of Arizona, we will continue to conduct community recruitment from Arizona so that we can examine treatment outcomes by state.

Second, the social media advertising and earned media (eg, interviews with news outlets) strategies were not as successful as in our pilot study. Therefore, we attempted to recruit using radio advertising. However, radio advertising was not effective either. We have engaged a recruiting firm to assist with community recruitment in Arizona. Despite these setbacks, we have consistently maintained our accrual goals. If needed, we have built in an additional 3 months in year 5 for continued recruitment.

Third, biochemical verification has been difficult for many participants because of inexperience using technology. Although study staff schedule Zoom sessions to *walk participants through* the data collection procedure, many of the participants find it difficult or impossible to complete the expired CO test. Therefore, self-reported 30-day abstinence is our primary outcome, and we will also examine 7-day self-reported and biochemically validated abstinence.

Finally, because the intervention is conducted entirely remotely with no person contact, we experience high attrition between randomization and the first treatment session. Therefore, we classify people as participants only after they have scheduled and completed their first treatment session, and all metrics regarding outcomes are reported for enrolled participants. Participants do not know to which condition they have been randomized until the first session. Therefore, we do not expect differential attrition before session 1 to cause bias while evaluating the intervention effects. Even if the differential attrition rate is not expected to relate to the intervention conditions, an inverse probability of attrition weighting approach [53] will be used to handle differential attrition rates and account for potential bias if attrition is indeed selective.

### Conclusions

The *Be Smoke Free* study is a first-of-its-kind RCT that incorporates GI into telephone-based tobacco cessation treatment. This study uses rigorous, transparent, and reproducible methods. The study protocols are based on strong theoretical frameworks and empirical evidence. We use best practices to address challenges in large studies with remote program delivery and data collection, well-established measures to reduce errors associated with self-report, successful recruitment and retention methods to reduce selection and nonresponse biases, and both intent-to-treat analysis and other methods to handle missing data.

Delivering a GI tobacco cessation intervention via telephone is a highly scalable novel integrative approach that may appeal to a broad range of smokers and increase the use of evidence-based tobacco treatment quitlines. This rigorous research has the potential for high overall impact. If successful, the *Be Smoke Free* program will have substantial benefits for the long-term health of people who use tobacco across the United States.

## Abbreviations

ASHLine: Arizona Smokers’ Helpline
BC: behavioral condition
CAB: community advisory board
CO: carbon monoxide
GI: guided imagery
GIC: guided imagery condition
HIPAA: Health Insurance Portability and Accountability Act
PI: principal investigator
RCT: randomized clinical trial
REDCap: Research Electronic Data Capture
T0: baseline
T1: 3 months after enrollment
T2: 6 months after enrollment

## Data Availability

Data from this study will be made available after the outcome paper is published or 6 months after the end of the study, whichever comes first.
